# Anticholinergic burden among in-patients: a cross-sectional study on prevalence, determinants, and impact on mortality in Ethiopia

**DOI:** 10.1177/20420986241259624

**Published:** 2024-06-14

**Authors:** Eyob Alemayehu Gebreyohannes, Wagaye Atalay Taye, Biniam Siyum Shibe, Emneteab Mesfin Ayele, Kenneth Lee, Segenet Bizuneh Mengistu, Roy Louis Soiza, Phyo Kyaw Myint, Ousman Abubeker Abdela

**Affiliations:** Quality Use of Medicines and Pharmacy Research Centre, UniSA Clinical and Health Sciences, University of South Australia, City East Campus, Frome Road, Adelaide, SA 5000, Australia; School of Allied Health, The University of Western Australia, Perth, WA, Australia; School of Medicine, Medical Sciences and Nutrition, University of Aberdeen, Aberdeen, UK; Department of Clinical Pharmacy, School of Pharmacy, University of Gondar, Gondar, Ethiopia; Department of Clinical Pharmacy, School of Pharmacy, University of Gondar, Gondar, Ethiopia; Department of Clinical Pharmacy, School of Pharmacy, University of Gondar, Gondar, Ethiopia; School of Allied Health, The University of Western Australia, Perth, WA, Australia; Department of Internal Medicine, School of Medicine, University of Gondar, Gondar, Ethiopia; School of Medicine, Medical Sciences and Nutrition, University of Aberdeen, Aberdeen, UK; School of Medicine, Medical Sciences and Nutrition, University of Aberdeen, Aberdeen, UK; School of Medicine, Medical Sciences and Nutrition, University of Aberdeen, Aberdeen, UK; Department of Clinical Pharmacy, School of Pharmacy, University of Gondar, Gondar, Ethiopia

**Keywords:** Anticholinergic burden, hospitalization, adult, Anticholinergic Cognitive Burden Score

## Abstract

**Background:**

Numerous studies report that anticholinergic burden (ACB) has been linked with several health consequences, including increased hospital admissions, prolonged hospitalization, and physical and cognitive impairment. However, low- and middle-income settings, as well as younger individuals, are underrepresented.

**Objectives:**

To assess the prevalence and determinants of ACB, and to assess the impact of ACB on in-hospital mortality among adult in-patients at University of Gondar Comprehensive Specialized Hospital (UOGCSH).

**Design:**

A cross-sectional study was conducted from June to August 2022 at UOGCSH among adult in-patients.

**Methods:**

A pre-tested questionnaire was utilized to collect data from patients and their corresponding medical charts. A consecutive sampling technique was used to select the participants. Descriptive statistics were used to summarize socio-demographic and clinical characteristics. Chi-squared, Fisher’s exact, and Wilcoxon rank sum tests, as appropriate, were used to determine associations between independent variables and ACB. Kaplan–Meier survival curve and Cox proportional hazards regression test were used to assess the impact of ACB on in-hospital mortality.

**Results:**

A total of 420 adult in-patients, median (interquartile range) age of 38 (26, 55) years, participated in this study. Over half (58.3%) were exposed to anticholinergic medicines, with a high ACB (⩾3) seen in 11.2% of participants. High ACB was associated with higher median number of medicines per patient (*p* = 0.003) higher median hospital length of stay (*p* = 0.033), and having mental and behavioral disorders (*p* < 0.001). No significant association was found between ACB and in-hospital mortality (log-rank test *p* = 0.26, Cox regression adjusted hazard ratio: 1.47, 95% CI: 0.335–6.453, *p* = 0.61).

**Conclusion:**

Among adult in-patients, a significant majority (58.3%) were subjected to medications possessing anticholinergic properties, with a noteworthy 11.2% of the study subjects exhibiting a high ACB. Participants with higher median length of hospital stay were more likely to have high ACB even in this relatively younger adult patient population.

## Plain language summary

Understanding the impact of medicines with anticholinergic properties on patients at University of Gondar Hospital

**Background:**

Anticholinergics refers to substances that block the action of the neurotransmitter acetylcholine in the body. Previous studies have shown that medicines exhibiting anticholinergic effects could lead to increased hospital admissions, longer hospital stays, and both physical and cognitive impairments.

**Objective:**

In this study, we aimed to assess how medicines exhibiting anticholinergic effects might affect patients in Ethiopian in-patient settings.

**Methods:**

We conducted a cross-sectional study from June to August 2022, collecting data from adult in-patients through a questionnaire and medical charts. We used a widely recognized tool called Anticholinergic Cognitive Burden Score to measure anticholinergic burden. We used statistical analyses to identify associations between the use of anticholinergic medicines and various factors, including the number of medicines per patient and the length of hospital stay. Additionally, we explored the impact of anticholinergic burden on in-hospital mortality.

**Results:**

Out of the 420 participants, 245 were exposed to medicines with anticholinergic properties. High anticholinergic burden was observed in 47 patients. Patients with mental and behavioral disorders were more likely to have high anticholinergic burden, while those with diseases of the digestive system were less likely. Moreover, a high anticholinergic burden was linked to a greater median number of medicines per patient and an extended median hospital length of stay. However, the study found no significant difference in in-hospital mortality between patients with high and low anticholinergic burden.

**Conclusion:**

The study highlights that a significant proportion of the participants were exposed to medicines with anticholinergic properties, and a notable percentage experienced a high anticholinergic burden. This burden was particularly associated with mental and behavioral disorders, the use of higher number of medicines, and longer hospital stay. Importantly, the research did not find a clear link between anticholinergic burden and in-hospital mortality after accounting for other factors.

## Introduction

Anticholinergics are class of medicines that are commonly prescribed for symptomatic management of different conditions such as Parkinson’s disease, muscle spasms, allergy, excessive gastric acid, nausea and vomiting, intestinal motility disorders, overactive bladder, and chronic obstructive pulmonary disease.^1,2^ They function by inhibiting the activity of the neurotransmitter acetylcholine in both the brain and peripheral tissues.^3^ This inhibitory mechanism leads to varying levels of anticholinergic activity in several commonly prescribed medicines from various pharmacological classes, contributing to anticholinergic burden (ACB) and resulting in minimal to severe adverse effects.^3,4^ ACB is the cumulative exposure of one or more medicines with potential anticholinergic activity. Current evidence shows that a higher score of ACB or prolonged exposure to a specific medicine with an anticholinergic effect has been linked to a greater risk of functional and cognitive decline, stroke, postoperative delirium, morbidity, and mortality.^4^

ACB is increasing globally and is becoming a major concern as it correlates with various short and long-term adverse clinical outcomes.^5–8^ Due to the widespread prescription of medicines with anticholinergic properties, its prevalence kept growing in various healthcare settings.^3^

Despite the growing interest in the negative clinical outcomes of anticholinergic drug use, low- and middle-income settings, as well as younger individuals, are underrepresented in the assessment of ACB and factors associated with ACB.^9^ No published studies were retrieved in Ethiopia focusing on ACB and its determinants in in-patients settings in the general population. To the best of the authors’ knowledge, this is the first study of its kind on ACB and its determinants among adult in-patients in this country. A better knowledge of the extent and the factors associated with the ACB would allow more effective and focused interventions to reduce the ACB whenever possible in vulnerable populations. Hence, this study had two aims: to assess the prevalence and determinants of ACB, and to assess the impact of ACB on in-hospital mortality among adult in-patients at a teaching hospital in Ethiopia.

## Materials and methods

### Study setting and period

The study was conducted within the in-patient wards of the University of Gondar Comprehensive and Specialized Hospital (UOGCSH). UOGCSH, a tertiary care facility, encompasses 960 beds and currently serves a population exceeding 13 million.^10^

### Study design and population

A cross-sectional study was conducted on adult patient who were admitted to surgical, psychiatric, internal medicine, and gynecological wards from 1 June to 30 August 2022. Discharge summaries were also reviewed for each study participants.

### Eligibility criteria

All adult patients aged 18 years and above, who were admitted to the surgical, gynecologic, psychiatric, and internal medicine wards at UOGCSH for any medical condition during the study period, were included. However, patients with severe communication disorders, those unwilling to provide written consent, individuals with unstable health status (those admitted to an intensive care unit), receiving palliative care, or having incomplete medical records were excluded from the study.

### Sample size determination and sampling technique

The sample size was determined by using a single population proportion formula with a 5% margin of error and 95% level of significance.^11^ Since there was no prior study in similar setting, a conservative value of 0.5 (50%) was taken for prevalence. After considering a 10% contingency, the final sample size was calculated to be 422. A consecutive sampling technique was used to recruit the study participants.

### Study variables

*Outcome variables*: ACB and in-hospital mortality.

*Independent variables*: Socio-demographic characteristics of the study participants include age, sex, residence, marital status, educational status, and socioeconomic class. (Occupation was used to classify socioeconomic class according to an occupation-based classification scheme.^12^ Social Class I consists of professionals, Class II includes managerial and technical occupations, Class III includes skilled workers, Class IV consists of partly skilled workers, and Class V comprises unskilled manual workers.) Other independent variables included clinical and medicine-related information such as ACB, comorbid conditions, Charlson Comorbidity Index (CCI) score, the number of medicines, specific wards, prior hospital admission in the past 30 days, and the length of hospital stay.

### Definitions of terms

ACB was defined as the cumulative exposure to anticholinergic effects resulting from taking one or more medicines possessing anticholinergic properties. It was calculated by using the Anticholinergic Cognitive Burden Score (ACBS).^3,13,14^

High and low ACBs were defined as cumulative scores of ⩾3 and <3,^15^ respectively, as measured by the ACBS.^14^

Polypharmacy was defined as the use of ⩾5 concurrent medicines.^16^

CCI is a method of categorizing comorbidities of patients by taking into accounts both the number and severity of 19 predefined comorbid conditions. Each condition was assigned a weight from 1 to 6, with total scores ranging from 0 to 37.^17^

### Data collection tools and procedures

The data collection tool was prepared based on a review of relevant literature. The tool consisted of three sections: (i) socio-demographic characteristics of the participants, (ii) clinical characteristics, and (iii) medicine-related information. Relevant data was obtained from medical records and interviews conducted with the study participants.

ACB was calculated according to the scoring system of medicines as described by Boustani *et al*.^14^ This system assigns a score of 0 (no known anticholinergic effect) to 3 (high anticholinergic effect) according to each medicines’ anticholinergic potential. An individual’s ACB score was calculated by summing the ACB scores assigned for each of the medicine taken by the patient. Medication, whether prescription or over-the-counter, is factored into ACB calculations throughout the hospitalization period. We include medications initiated either before admission (provided they are taken during the hospital stay) or during the hospital stay itself.

### Data quality management

Data were collected by a clinical pharmacy postgraduate student who were properly trained on the contents of the instrument, data collection methods, data handling and documentation, and ethical issues. To verify the accuracy of each patient’s medication list, the data collector carefully cross-checked the information from the patients’ medical records with input from patients, caregivers, treating nurses, and also conducted physical checks of the medications at the patient’s bedside. The local principal investigator (OAA) supervised the data collection process. A pre-test was conducted on 5% of the total sample size, involving randomly selected patients from eligible wards. The data from these patients were subsequently excluded from the final analysis. The collected data were checked for completeness, accuracy, and consistency on a daily basis during the data collection period. The reporting of this study conforms to the Strengthening the Reporting of Observational Studies in Epidemiology (STROBE) guidelines.^18^

### Data entry and analysis

The collected data were exported to R (version 4.2.2; R Foundation for Statistical Computing, Vienna, Austria) for analysis. Descriptive statistics including median, interquartile range (IQR), frequencies, and percentages were used to summarize socio-demographic and clinical data. Chi-squared, Fisher’s exact, and Wilcoxon rank sum tests, as appropriate, were used to determine associations between independent variables and ACB as a dichotomized outcome (high *versus* low).

Kaplan–Meier survival curve and Cox proportional hazards regression test were used to assess the impact of ACB on in-hospital mortality. The log-rank test was used to determine statistical significance in survival curves between high and low ACB. In the Cox proportional hazards regression models, we conducted unadjusted univariate analysis with ACB as the only predictor variable (model A), and made sequential adjustments for age, sex, medicine number, and prior admission in the past 30 days. Independent variables, with the exception of sex, that had *p* values <0.05 on univariate analysis were included in the final model. Scaled Schoenfeld residuals and Martingale residuals were used to assess the proportionality and linearity assumptions of the Cox proportional hazard regression, respectively. The Akaike information criterion (AIC) was used to select the best model. In all statistical analysis, a *p* value of <0.05 was considered statistically significant.

## Results

### Socio-demographic characteristics of the study participants

A total of 420 patients participated in the study, 55% of whom were female. Age of participants ranged from 19 to 90 years, with a median (IQR) of 38 (26, 55). Approximately two-thirds (67.1%) of the participants were married, and about half of them (51.7%) were rural dwellers. More than two-thirds (78.6%) of the participants were unskilled manual workers and nearly half (47.6%) of them did not have a formal education. Most participants (62.6%) were from internal medicine ward, and 14% of the participants had prior hospital admission within the preceding 30 days. Infectious and parasitic diseases (46.2%), diseases of the circulatory system (36.7%), and diseases of the digestive system (19.3%) were the most common co-morbidities. The median (IQR) number of medicines per patient was 4 (3, 5), and168 (40%) of the participants had polypharmacy. A total of 13 (3.1%) participants died during their hospital stay (Table 1).

**Table 1.** Socio-demographic profile of adult in-patient study participants at UOGCSH (*n* = 420).

Characteristics	Frequency (%)
Sociodemographic characteristics	
Age – median (IQR)	38 (26, 55)
Sex – *n* (%)	
Male	189 (45%)
Female	231 (55%)
Residency – *n* (%)	
Urban	203 (49.3%)
Rural	217 (51.7%)
Marital status – *n* (%)	
Married	282 (67.1%)
Never married/divorced/widowed	138 (32.9%)
Education – *n* (%)	
No formal education	200 (47.6%)
Primary education	121 (28.8%)
Secondary or tertiary education	99 (23.6%)
Socioeconomic class – *n* (%)	
Class I to IV	90 (21.4%)
Class V	330 (78.6%)
Smoking status – *n* (%)	
Non-smoker	408 (97.1)
Past/current smoker	12 (2.9)
Clinical characteristics	
Medical specialty (wards)	
Internal medicine	263 (62.6%)
Psychiatry	25 (6%)
Surgical/gynecology	132 (31.4%)
Charlson Comorbidity Index – median (IQR)	1 (0, 3)
Co-morbidities	
Infectious and parasitic diseases	194 (46.2%)
Diseases of the circulatory system	154 (36.7%)
Diseases of the digestive system	81 (19.3%)
Diseases of the blood	57 (13.6%)
Endocrine, nutritional and metabolic diseases	46 (11%)
Diseases of the genitourinary system	46 (11%)
Mental and behavioral disorders	26 (6.2%)
Neoplasms	18 (4.3%)
Diseases of the respiratory system	12 (2.9%)
Number of medicines per patient – median (IQR)	4 (3, 5)
Poly-pharmacy – *n* (%)	
Yes	168 (40%)
Prior admission within 30 days – *n* (%)	
Yes	59 (14%)
In-hospital mortality – *n* (%)	
Yes	13 (3.1%)
Hospital length of stay – median (IQR)	12 (8, 18)

The same patient may have several reason for hospitalization and co-morbidities, the percentage may greater than 100%.

IQR, interquartile range; UOGCSH, University of Gondar Comprehensive and Specialized Hospital.

### Prescribing pattern of anticholinergic medicines

**Figure 1.**
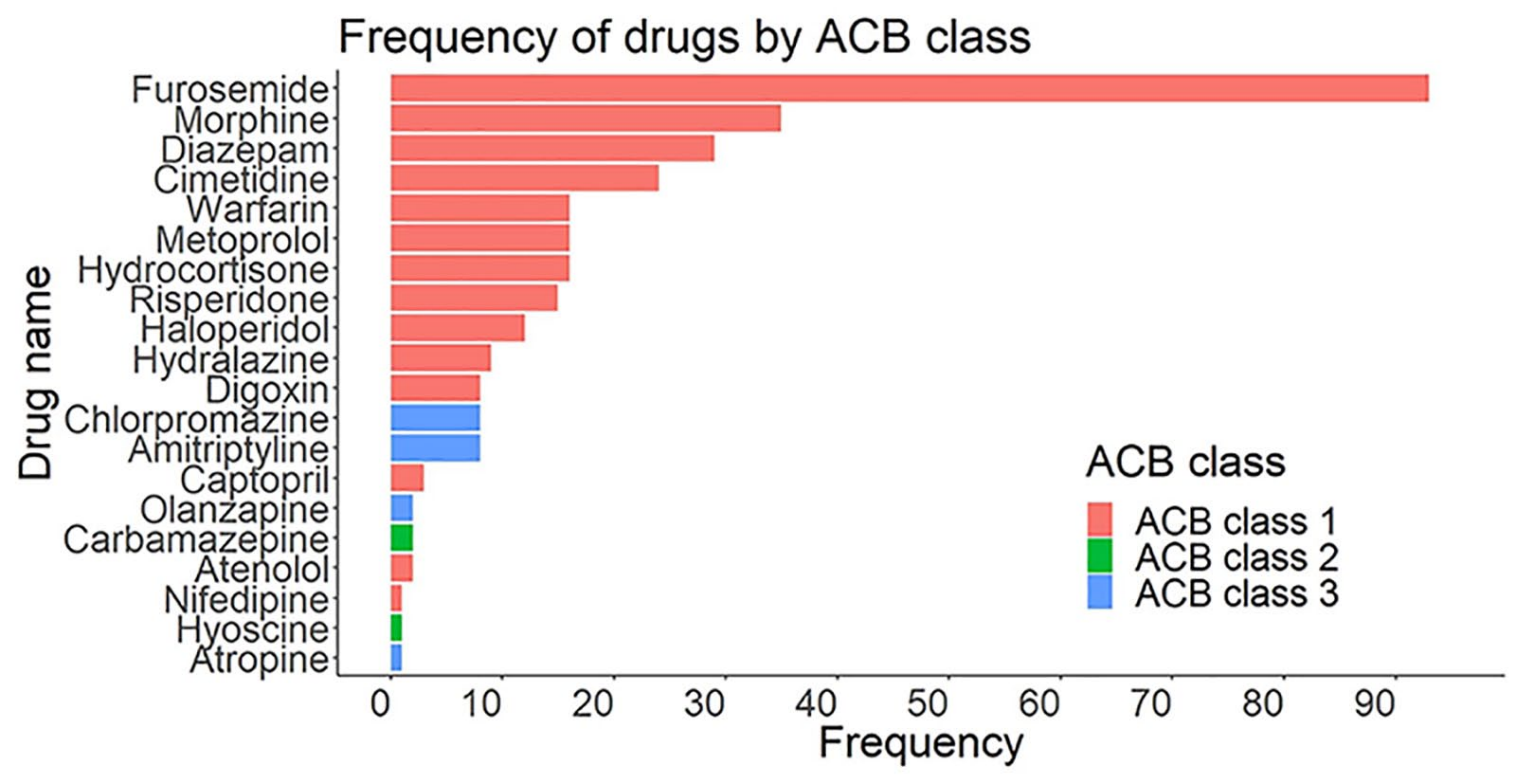
Frequency of medicines with anticholinergic properties prescribed to study participants at UOGCSH (*n* = 420). UOGCSH, University of Gondar Comprehensive and Specialized Hospital.

More than half (58.3%) of the study participants were exposed to at least one medicine with anticholinergic activity (total ACB score ⩾1), and the highest ACB score was 7 (*n* = 1). A high ACB was found in 11.2% of participants. The most frequently prescribed individual medicines with anticholinergic properties were furosemide (*n* = 93), followed by morphine (*n* = 40), and diazepam (*n* = 31). Most medicines identified to have anticholinergic properties had an ACB score of 1 (92.0%), followed by a score of 3 (7.4%) and a score of 2 (0.6%) (Figure 1).

### Factors associated with high ACB

Tables 2 and 3 provide information on associations between the socio-demographic and clinical characteristics of study participants with ACB. None of the socio-demographic characteristics showed statistically significant associations with ACB (Table 2). High ACB was associated with higher median number of medicines per patient (*p* = 0.003) and long median hospital length of stay (*p* = 0.033). In addition, patients who have mental and behavioral disorders (*p* < 0.001) were more likely to have high ACB, whereas patients with diseases of the digestive system (*p* = 0.004) were less likely to have high ACB (Table 3).

**Table 2.** Associations between socio-demographic characteristics of study participants and ACB at UOGCSH (*n* = 420).

Characteristics	Low ACB (ACB score <3), *n* = 383	High ACB (ACB score ⩾3), *n* = 37	*p* Value
Age – median (IQR)	38 (26, 55)	40 (30, 57)	0.475^c^
Sex – *n* (%)			
Male	172 (44.9%)	17 (46.0%)	0.999^a^
Female	211 (55.1%)	20 (54.0%)	
Residency – *n* (%)			
Urban	185 (48.3%)	18 (48.7%)	0.999^a^
Rural	198 (51.7%)	19 (51.4%)	
Marital status – *n* (%)			
Married	258 (67.4%)	24 (64.9%)	0.900^a^
Never married/divorced/widowed	125 (32.6%)	13 (35.1%)	
Education – *n* (%)			
No formal education	178 (46.5%)	22 (59.5%)	0.300^a^
Primary education	112 (29.2%)	9 (24.3%)	
Secondary or tertiary education	93 (24.3%)	6 (16.2%)	
Socioeconomic class – *n* (%)			
Class I to IV	80 (20.9%)	10 (27.0%)	0.510^a^
Class V	303 (79.1%)	27 (73.0%)	
Smoking status – *n* (%)			
Non-smoker	371 (96.9%)	12 (100.0%)	0.611^b^
Past/current smoker	37 (3.1%)	0 (0.0%)	

a Chi-squared test.

b Fisher’s exact test.

c Wilcoxon rank sum test.

ACB, anticholinergic burden; IQR, interquartile range; UOGCSH, University of Gondar Comprehensive and Specialized Hospital.

**Table 3.** Associations between clinical characteristics of study participants and ACB at UOGCSH (*n* = 420).

Characteristics	Low ACB (ACB score <3), *n* = 383	High ACB (ACB score ⩾3), *n* = 37	*p* Value
Medical specialty (wards)			
Internal medicine	241 (62.9%)	22 (59.5%)	0.812^a^
Surgical/psychiatry/gynecology	142 (37.1%)	15 (40.5%)	
Charlson Comorbidity Index – median (IQR)	1 (0, 3)	1 (1, 2)	0.036^c^
Comorbidity – *n* (%)			
Infectious and parasitic diseases	179 (46.7%)	15 (40.5%)	0.583^a^
Neoplasms	18 (4.7%)	0 (0%)	0.388^b^
Diseases of the circulatory system	141 (36.8%)	13 (35.1%)	0.981^a^
Endocrine, nutritional, and metabolic diseases	41 (10.7%)	5 (13.5%)	0.582^b^
Diseases of the digestive system	80 (20.9%)	1 (2.7%)	0.004^b^
Diseases of the respiratory system	11 (2.9%)	1 (2.7%)	0.999^b^
Diseases of the genitourinary system	41 (10.7%)	5 (13.5%)	0.582^b^
Mental and behavioral disorders	14 (3.7%)	12 (32.4%)	<0.001^b^
Diseases of the blood	55 (14.4%)	2 (5.4%)	0.205^b^
Number of medicines per patient – median (IQR)	4 (3, 5)	5 (4, 7)	0.003^c^
Polypharmacy – *n* (%)			
Yes	148 (38.6%)	20 (54.1%)	0.099^a^
Prior admission within 30 days – *n* (%)			
Yes	56 (14.6%)	3 (8.33)	0.432^a^
In-hospital mortality – *n* (%)			
Yes	10 (2.6%)	3 (8.1%)	0.097^b^
Hospital length of stay – median (IQR)	12 (8, 17)	16 (10, 22)	0.033^c^

a Chi-squared test.

b Fisher’s exact test.

c Wilcoxon rank sum test.

ACB, anticholinergic burden; IQR, interquartile range; UOGCSH, University of Gondar Comprehensive and Specialized Hospital.

### High ACB and in-hospital mortality

**Figure 2.**
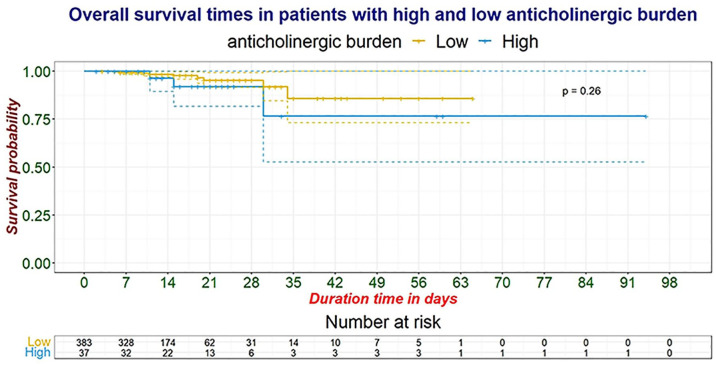
Survival analysis: Kaplan–Meier curve comparing in-hospital survival in patients with high and low ACB at UOGCSH (*n* = 420). The dotted lines represent the 95% confidence intervals. ACB, anticholinergic burden; UOGCSH, University of Gondar Comprehensive and Specialized Hospital.

The overall 12th-day (i.e. the median number of hospital length of stay) survival (95% CI) was 98% (97–100%). The corresponding figures for patients with high and low ACB were 96% (89–100%) and 98% (97–100%), respectively. However, the Kaplan–Meier survival curve revealed no differences (log-rank test: *p* = 0.26) in in-hospital mortality between patients with high and low ACB (Figure 2).

Table 4 represents the hazard ratios (HRs) and their corresponding 95% CI depicting the risk of in-hospital mortality for individuals with high ACB compared to those with low ACB. After adjusting for age, sex, numbers of medicines, prior admission in the past 30 days, and CCI, the HR (95% CI) for in-patient mortality in patients with high ACB was 1.47 (0.335–6.453) times higher compared to those with low ACB; however, this effect was not statistically significant (*p* = 0.61).

**Table 4.** Cox proportional hazards regression analysis: in-hospital mortality in patients with high and low ACB at UOGCSH (*n* = 420).

Model	Variable in the model	OR (95% CI)	*p* Value	AIC
A	ACB	2.089 (0.569–7.673)	0.267	128.9
B	A + Age	1.781 (0.484–6.548)	0.385	124.5
C	B + Sex	1.789 (0.486–6.585)	0.381	126.5
D	C + Medicine number	0.998 (0.235–4.242)	0.998	121.1
E	D + Prior admission in the past 30 days	1.285 (0.295–5.591)	0.739	121.1
F	E + CCI	1.470 (0.335–6.453)	0.610	116.0

ACB, anticholinergic burden; AIC, Akaike information criterion; CCI, Charlson Comorbidity Index; OR, odds ratio; UOGCSH, University of Gondar Comprehensive and Specialized Hospital.

## Discussion

In this study, we aimed to investigate the prevalence of ACB among hospitalized adult patients (aged 18 and over) at a teaching hospital in Ethiopia. We also sought to identify factors associated with high ACB and its potential impact on in-hospital mortality. The key findings of our research indicate a substantial prevalent use of medicine with anticholinergic properties even in this relatively young patient population (median age of 38 years), with 58.3% of participants exposed to at least one medicine with anticholinergic activity. Notably, a high ACB was found in 11.2% of participants. To the best of our knowledge, this study is among the first to comprehensively explore the epidemiology of ACB in a low-income setting in a relatively younger patient population, shedding light on an underexplored area of research in the Ethiopian healthcare context.

The prevalence rates, particularly the proportion of patients with a high ACB, may serve as a valuable reference for healthcare providers and clinical researchers in Ethiopia and other similar settings. Our study findings are novel as it encompassed adult patients across all age groups, whereas most previous studies focused only on older individuals. The prevalence of high ACB observed in our findings was comparatively lower than that reported in several prior studies, which could partly be explained by the inclusion of younger adults. For instance, a high ACB was reported in 49.1% of hospitalized older adults in Korea.^19^ Another study reported a prevalence of 19.1%, with 90% of the patients being aged 65 years or older.^20^ Similarly, a study from Turkey also reported a high ACB prevalence of 21%.^21^

Our study identified factors associated with ACB, providing insights into the determinants of ACB in a hospital setting. Notably, the median number of medicines per patient and a prolonged hospital length of stay were positively associated with ACB. ACB is a cumulative outcome resulting from the use of one or more medicine with anticholinergic effects. In addition to the use of medicines with high ACB scores, high ACB may also result from the concomitant use of several low-risk medicines.^22^ Hence, it is expected that there is a positive correlation between the number of medicines an individual takes and the resultant ACB. This is in agreement with previous reports.^23,24^

Consistent with our study’s findings, previous studies have reported a statistically significant positive association between a higher ACB and an increased length of hospital stay.^15,19^ Furthermore, our study sheds light on a critical insight, highlighting the significance of ACB even among younger adults, beyond its traditionally recognized impact on older populations. Moreover, a positive correlation was observed between the occurrence of mental and behavioral disorders and ACB. This relationship can be explained by the fact that certain medicines employed for treating these disorders are recognized for their anticholinergic properties. Furthermore, it is common for anticholinergic medicines to be prescribed simultaneously to mitigate specific side effects associated with these drugs.^25^ These findings suggest the need for tailored prescribing practices, especially in patients with multiple medicines or specific medical conditions. Understanding these determinants is crucial for developing targeted interventions to reduce ACB and improve patient outcomes.

Analysis of in-hospital mortality in relation to ACB revealed no statistically significant association. However, previous studies reported that compared to no-to-low ACB, exposure to high ACB was associated with up to threefold increase in the risk of in-hospital mortality in older hospitalized patients aged ⩾65 years.^15,19^ The contrast with our own results could be due to our study recruiting a much younger population, suggesting that high ACB poses a significant mortality risk to older people only. Future studies may delve into specific subgroups or conditions where the impact of ACB on mortality could be more pronounced, guiding clinical decision-making and resource allocation. Lastly, artificial intelligence and/or machine-learning applied to large and complex data pools could improve prognostication.^26^

### Strengths and limitations of the study

This study contributes valuable insights into the prevalence and determinants of ACB in a low-income setting, but it is not without limitations. Although a sample size of 420 participants sufficed to determine the prevalence of ACB in the study population, it might not be sufficient for identifying important determinants of ACB or demonstrating its impact on in-hospital mortality, as indicated by previous studies employing larger sample sizes or more homogenous study population.^15,19^ In addition to the age-related considerations, it is important to highlight that our study exclusively examined a hospitalized patient population, limiting the extrapolation of findings to community or non-hospital settings. Nevertheless, the high prevalence of ACB observed in our study raises questions about the association between ACB and the risk of hospitalization. Furthermore, the cross-sectional nature of the study limits our ability to establish causation. As an epidemiological examination, we may not be able to account for all known or unknown confounders and residual confounding even after adjusting for potential confounders. In the absence of a universally accepted tool for measuring ACB, we relied on an expert-based scale that is commonly used in clinical settings.^14^ As such, we did not consider the duration of drug use, dosages, and the impact of recently discontinued medications in calculating ACB scores. Lastly, although it is possible that some of the hospitalizations in our study were caused by symptoms of high ACB, we did not collect data on specific anticholinergic symptoms to verify this. Despite these limitations, our study lays the foundation for future investigations in similar settings.

## Conclusion

In conclusion, our investigation underscores the prevalent exposure to medicines with anticholinergic properties among hospitalized adults, affecting 58.3% of admitted patients. Notably, our study reveals that high ACB was identified in 11.2% of participants. Factors such as a higher median number of medicines, extended hospital stays, and the presence of mental and behavioral disorders were positively associated with the ACB. Interestingly, our findings did not establish a significant association between ACB and in-hospital mortality, possibly because our population was relatively younger than others which reported such an association. This prompts a call for future comprehensive large-scale prospective cohort studies at the population level to evaluate trends and associated risks with anticholinergic use, stratified by age groups.

## Declarations

**Ethics approval and consent to participate:** The Institutional Ethical Review Board of the University of Gondar granted ethics approval for conducting this research (VP/RTT/05/359/2021). Ethics approval was also obtained from the sponsor of the project, University of Aberdeen School of Medicine, Medical Sciences and Nutrition Ethics Review Board (SERB) (CERB/2021/7/2108). Permission to collect data was also acquired from the UOGCSH Clinical Directorate. Written and verbal consent were obtained from respondents after explaining the study’s purpose. Participant’s confidentiality was ensured by refraining from recording their identifiers on the questionnaire.

**Consent for publication:** Not applicable.

**Availability of data and materials:** The datasets used for the current study are available from the corresponding author on reasonable request.
